# Feasible and Rapid Screening of *IDH1/2* and *FLT3-TKD2* Mutations by High-Resolution Melting for Patients with Acute Myeloid Leukemia

**DOI:** 10.3390/diagnostics15101230

**Published:** 2025-05-14

**Authors:** José Vicente Gil, Sandra de las Heras, Alberto Miralles, Claudia Sargas, Marta Llop, Rebeca Rodríguez-Veiga, Laura Torres-Miñana, Blanca Boluda, Isabel Cano-Ferri, Evelyn Acuña-Cruz, Irene Navarro, Pilar Lloret-Madrid, Pau Montesinos, Eva Barragán

**Affiliations:** 1 Accredited Research Group on Hematology, Instituto de Investigación Sanitaria la Fe, 46026 Valencia, Spain; jose_gil@iislafe.es (J.V.G.);; 2 Molecular Biology Unit, Clinical Analysis Service, Hospital Universitario y Politécnico la Fe, 46026 Valencia, Spain; 3 Centro de Investigación Biomédica en Red de Cáncer, CIBERONC Instituto de Salud Carlos III, 28029 Madrid, Spain; 4 Hematology Department, Hospital Universitario y Politécnico la Fe, 46026 Valencia, Spain

**Keywords:** acute myeloid leukemia (AML), high resolution melting (HRM), targeted therapies, *IDH1/2*, *FLT3-TKD2*, mutation screening

## Abstract

**Background**: In recent years, numerous recurrently mutated genes have been identified in acute myeloid leukemia (AML), some of which, such as *FLT3* and *IDH1/2*, serve as therapeutic targets, offering new treatment options. Rapid mutational analysis is crucial for timely and optimal therapy selection. This study aims to develop and validate a rapid, cost-effective, and sensitive screening method for detecting *IDH1*, *IDH2*, and *FLT3*-TKD2 mutations using polymerase chain reaction (PCR) and high-resolution melting curve analysis (HRM). **Methods**: A PCR-HRM assay was developed to simultaneously detect mutations in *IDH1*, *IDH2*, and *FLT3*-TKD2. The method was applied to a cohort of 1363 AML patients, and its performance, including turnaround time, was evaluated through comparison with next-generation sequencing (NGS) results. **Results**: The PCR-HRM method demonstrated a positive percent agreement of 98%, 98%, and 92% for *IDH1*, *IDH2*, and *FLT3-TKD2*, respectively, and a negative percent agreement of 100% for all three genes compared to NGS. No false positives were observed, and false negatives were detected in less than 1% of cases, mostly in *FLT3*-TKD2, all occurring below the established limit of detection. The turnaround time and cost of PCR-HRM were significantly lower than those of NGS. **Conclusions**: This method offers a highly sensitive, specific, and time-efficient approach for the simultaneous detection of *IDH1*, *IDH2*, and *FLT3*-TKD2 mutations in AML patients. Its rapid turnaround time and cost-effectiveness make it a valuable tool for routine clinical screening, facilitating timely and targeted treatment decisions.

## 1. Introduction

Acute myeloid leukemia (AML) is a heterogeneous disease characterized by a wide spectrum of molecular alterations [1]. Advances in molecular knowledge of AML have provided relevant data about new biomarkers for diagnosis, risk stratification, and targeted therapy selection [2]. This is exemplified by targeted small-molecule inhibitors against mutant *IDH1/2* and *FLT3*, which are currently in clinical development for the treatment of newly diagnosed, relapsed, and refractory AML patients [3,4].

Isocitrate dehydrogenases are homodimeric enzymes encoded by *IDH1* and *IDH2* genes, which develop their function mainly in the citric acid cycle. They regulate some cellular processes, including hypoxia adaptation, histone demethylation, and other DNA modifications [5]. Mutations in *IDH1* and *IDH2* are reported in 5 to 15% of AML cases, respectively, being p.Arg132X (NM_005896.4:*IDH1*) and p.Arg140X, p.Arg172X (NM_002168.4:*IDH2* the most frequent alterations) [6,7]. These mutations result in D-2-hidroxyglutarate overproduction, which interferes with cellular metabolism regulation [7]. Although clinical outcomes remain controversial, the emerging therapeutic use of targeted inhibitors has made their detection essential in AML, as they might improve the prognosis of *IDH1/2*-mutated AML patients [8].

FLT3 is a class III tyrosine kinase receptor that plays an important role in myeloid lineage development. The interaction of FLT3 with FLT3 ligand produces a conformational change leading to the autophosphorylation of the tyrosine kinase domains [9]. Phosphorylated FLT3 promotes the activation of RAS/MAPK, AKT/PI-3K, and JAK/STAT5a pathways [10,11]. *FLT3* mutations appear in 25–35% of AML cases with either internal tandem duplications (*FLT3-ITD*) within the juxta membrane domain, or missense mutations affecting the second tyrosine kinase domain (*FLT3-TKD2*) [12]. Although the impact on survival of *FLT3-TKD* is not as detrimental as *FLT3-ITD*, mutational screening of this domain remains crucial for therapy selection, as the vast majority of tyrosine kinase inhibitors (TKI) are able to block FLT3 activity through competition with ATP at the FLT3-TKD site [13].

Various methods have been used to detect *IDH1/2* and *FLT3* mutations in AML patients, such as direct sequencing, Restriction Fragment Length Polymorphism-Polymerase Chain Reaction (RFLP-PCR) or, recently, next generation sequencing (NGS) [14,15]. However, these methods are expensive, time-consuming, and some of them are not able to detect these mutations in samples with low mutational burden. The current scenario of AML management highlights the need for rapid molecular assays to effectively screen relevant biomarkers. The European LeukemiaNet (ELN) recommendations stress the importance of identifying actionable genes as quickly as possible, ideally within 3 to 5 days. However, the cost and turnaround time of techniques like NGS can pose significant challenges, potentially delaying the initiation of treatment [16,17,18]. In this study, we aimed to develop a novel method based on real-time PCR and high-resolution melting curve analysis (HRM) to simultaneously detect *IDH1/2* and *FLT3-TKD2* alterations in AML patients, using Sanger sequencing as confirmation. We compared the results with those obtained by NGS, in order to assess the clinical applicability of this rapid and cost-effective method. The assay has a technical hands-on time of less than 2 h, a detection limit (LOD) of 5%, and has been successfully validated on over 1000 samples, demonstrating a median turnaround time (sample reception to result validation) of 2 days. Its speed and affordability make it accessible to most laboratories.

## 2. Materials and Methods

### 2.1. Clinical Samples

The Molecular Biology Unit of the Hospital Universitari i Politècnic La Fe serves as a national reference laboratory in Spain within the framework of the Programa Español para Tratamientos en Hematología (PETHEMA group) [19]. This laboratory plays a crucial role in the molecular characterization of acute myeloid leukemia (AML), ensuring standardized and high-quality genetic analyses that contribute to both clinical decision-making and research advancements. Between April 2016 and September 2021, we consecutively analyzed 1163 patients (median age: 66 years; range: 1–98 years) diagnosed with de novo or refractory/relapsed AML and treated according to PETHEMA group recommendations for both fit and unfit patients. A total of 1054 bone marrow (BM) and 309 peripheral blood (PB) samples were collected for *IDH1/2* screening, and 1125 BM and 301 PB samples for *FLT3-TKD2* analysis. All samples were obtained in EDTA-containing Vacutainer^®^ tubes.

These analyses were performed as part of the routine diagnostic workflow aimed at providing a rapid and standardized molecular characterization of AML at diagnosis and relapse. All patients provided written informed consent before sample collection, in accordance with the ethical guidelines outlined in the Declaration of Helsinki and the principles set forth in the Declaration of Human Rights. The study was approved by the institutional ethics committee, ensuring compliance with ethical and legal requirements for biomedical research.

AML subtypes at diagnosis were classified according to the French-American-British (FAB) and the World Health Organization (WHO) classifications [18]. Additionally, conventional cytogenetics was performed using standard banding techniques, and cytogenetic risk was assessed following the Medical Research Council (MRC) recommendations [20]. These analyses were critical for integrating molecular and cytogenetic information into risk stratification models, which guide treatment decisions and prognosis assessment.

Patients were included if they had a confirmed diagnosis of AML according to WHO criteria and sufficient sample material for molecular testing. Exclusion criteria included inadequate sample quality.

### 2.2. DNA Extraction

Genomic DNA was extracted from 400 µL of BM or 800 µL of PB using the QIAsymphony DNA Midi Kit (Qiagen, Hilden, Germany) on the QIAsymphony SP Automated Extraction Instrument (Cat 9001297, Qiagen, Hilden, Germany), following manufacturer’s protocol. This automated extraction system ensures high reproducibility and minimizes the risk of contamination, which is particularly relevant for downstream applications such as high-resolution melting (HRM) analysis and next-generation sequencing (NGS).

The quality and concentration of the extracted DNA were assessed using the NanoDrop 2000^®^ spectrophotometer (NanoDrop Technologies Inc., Wilmington, DE, USA). Samples with suboptimal purity (A260/A280 ratio outside the expected range of 1.8–2.0) or insufficient concentration were either re-extracted or subjected to additional purification steps using the MinElute Reaction Cleanup Kit (Qiagen) according to manufacturer’s recommendations. The extracted DNA was subsequently stored at −20 °C until further analysis.

### 2.3. PCR-HRM

PCR amplification was performed using 40 ng of total genomic DNA per reaction. The reaction mixture contained 0.3 µM of each primer, previously described by Tefferi et al. [21] and Yamamoto et al. [22], and synthesized by TIB MOLBIOL (Berlin, Germany), along with 3 mM of MgCl_2_ and 1× LightCycler^®^ 480 High Resolution Melting (HRM) Master Mix (Cat. 0909631001, Roche Molecular Biochemicals, Mannheim, Germany). All reactions were performed in singleplex format in a final volume of 10 µL. Thus, four separate reactions were required for each sample to evaluate the mutational status of *IDH1* (codon 132), *IDH2* (codons 140 and 172), and *FLT3-TKD2*. The PCR protocol consisted of an initial denaturation step at 94 °C for 2 min, followed by 55 amplification cycles, each including denaturation at 94 °C for 10 s, annealing at 55 °C for 10 s, and extension at 72 °C for 20 s. A final extension step was performed at 72 °C for 5 min to ensure complete amplification.

Following PCR amplification, the HRM analysis was conducted to assess DNA melting profiles. The HRM program included a denaturation step at 95 °C for 1 min, followed by a cooling step at 40 °C for 1 min. The melting curve was generated by gradually increasing the temperature from 65 °C to 95 °C at a rate of 0.02 °C per second, with 25 fluorescence acquisitions per degree. To ensure high-quality and reproducible results, samples were considered valid only if they met the following quality control parameters: a quantification cycle (Cq) below 35, a fluorescence signal above 20 units, and a single melting peak at the expected temperature, which ensured the absence of nonspecific amplification. Interpretation of the HRM curves was based on deviation from the wild-type melting profile: samples showing a distinct melting curve shift or altered curve shape compared to the wild-type reference were classified as positive; samples with identical melting profiles to the wild-type control were considered negative; and those with ambiguous or low-quality curves were classified as non-assessable.

Melting curve analysis was performed using the Gene Scanning software integrated into the LightCycler^®^ 480 Instrument (Roche Diagnostics, Roche Instrument Center AG, Rotkreuz, Switzerland). Based on HRM profiles, samples were classified as positive, negative, or non-assessable.

### 2.4. Analytical Performance of PCR-HRM

To assess assay precision, we evaluated repeatability (intra-assay precision) and reproducibility (inter-assay precision) using positive DNA samples with known mutations in *IDH1*, *IDH2*, and *FLT3-TKD2*.

To assess the limit of detection (LOD), a previously characterized positive sample showing a 30% variant allele frequency (VAF) was serially diluted with wild-type DNA to create a decreasing VAF gradient for *IDH1*, *IDH2*, and *FLT3-TKD2*.

### 2.5. Sanger Sequencing

To confirm and further characterize the alterations identified by PCR-HRM, direct Sanger sequencing was performed on positive samples. PCR products were first purified using the ExoSAP-IT™ PCR Product Cleanup Reagent (Thermo Fisher Scientific, San Francisco, CA, USA) to remove excess primers and nucleotides. The purified amplicons were then subjected to sequencing reactions using the BigDye™ Terminator Cycle Sequencing Kit (Thermo Fisher Scientific), following the manufacturer’s protocol. Sequencing products were analyzed using an automated capillary electrophoresis system to determine the exact nucleotide change.

### 2.6. Next Generation Sequencing (NGS)

Next-generation sequencing (NGS) was performed on all samples on the Ion Torrent platform using the Oncomine Myeloid Research Assay panel (Cat A36941, Thermo Fisher Scientific). Library and template preparation were carried out automatically with the Ion Chef™ Instrument (Thermo Fisher Scientific) using the Ion AmpliSeq™ Kit for Chef DL8 (Cat A29024, Thermo Fisher Scientific) and Ion 510™ and Ion 520™ and Ion 530™ Kit—Chef (Cat A34461, Thermo Fisher Scientific), respectively. The library kit allows the simultaneous preparation of 8 libraries from 10 ng of DNA per patient. Libraries were loaded into an Ion 530 chip (Thermo Fisher Scientific) and sequenced on an Ion S5 sequencer (Thermo Fisher Scientific). Uniformity > 90% and a mean coverage > 500× were established as the minimum quality parameters. VAF cut-off was set at 1%.

### 2.7. Turnaround Times

Turnaround time (TAT) was defined as the interval from sample reception to result validation, including all pre-analytical, analytical, and post-analytical steps. This parameter was recorded for each method and expressed as the median time in days. The analysis considered potential workflow variations due to factors such as instrument availability and manual versus automated steps.

Cost analysis was performed based on the direct expenses associated with each method, focusing on the reagents and consumables required per sample. Costs related to instrumentation depreciation, maintenance, and personnel salaries were not included in this analysis, as these factors can vary significantly across different laboratories and healthcare systems.

## 3. Results

### 3.1. Study of High-Resolution Melting

**Figure 1:**
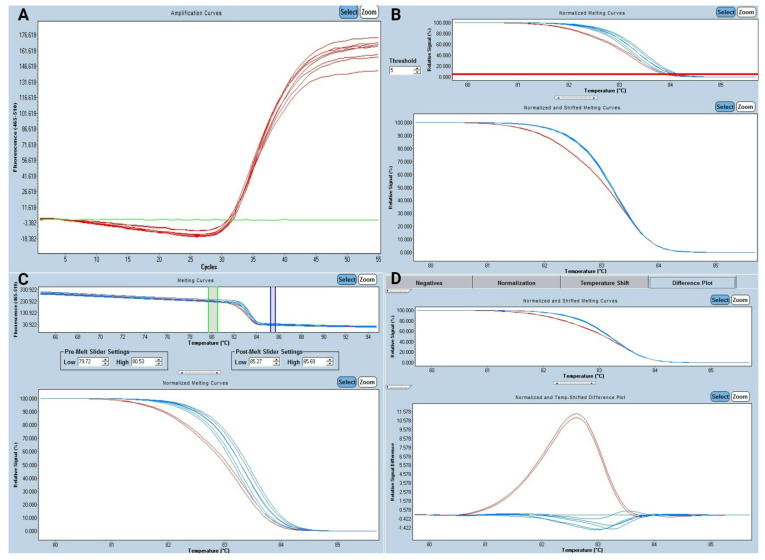
Different steps for the mutational detection in *IDH1*, *IDH2*, and *FLT3-TKD2* using Gene Scanning software: amplification curves show uniform amplification of all samples for *IDH1* screening (**A**). Normalized melting curves for *IDH1* detection show different groups due to the complementarity between both strands (**B**). Temperature shift using a 5% threshold (**C**). Difference plot separates both groups for *IDH1* screening (blue curves correspond to wild-type samples and red curves indicate positive samples) (**D**).

A total of 1363 samples from 1096 patients were screened for *IDH1* and *IDH2* exon 4 mutations, and 1426 samples from 1163 patients for *FLT3-TKD2*. Real-time PCR analysis revealed a mean Cq of 30 for all amplicons, including both wild-type and variant sequences of *IDH1*, *IDH2*, and *FLT3-TKD2*, when using 40 ng of DNA per reaction (Figure 1A). This consistency in Cq values suggests similar amplification efficiencies across the three genes. Melting analyses revealed a clear difference between the wild-type sample group and the potential positive samples (Figure 1B,C). A difference plot analysis was carried out to increase the difference between positive and negative samples using a wild-type control as a baseline (Figure 1D).

Difference plot curves were classified as positive for *IDH1*, in 117/1363 (8.58%) samples, 168/1363 (12.33%) for *IDH2* (146/168 p.Arg140X and 22/168 p.Arg172X), and 78/1426 (5.47%) for *FLT3-TKD2*. According to the established quality parameters, 23 samples (1.69%) were not assessable for *IDH1* or *IDH2*, and 27 (1.89%) for *FLT3-TKD2*, due to insufficient amplification caused by low DNA concentration, which resulted in poor separation of melting curves and the appearance of nonspecific melting peaks.

### 3.2. Analytical Performance for HRM Analysis

For repeatability assessment, three independent replicates of mutated and wild-type samples were run in the same assay on the same day. The melting profiles were consistent across replicates, and no variability in mutation detection was observed. For reproducibility, the same samples were analyzed on three different days by two independent operators. In all cases, the melting curves and mutation classification results were concordant, demonstrating a high reproducibility.

**Figure 2:**
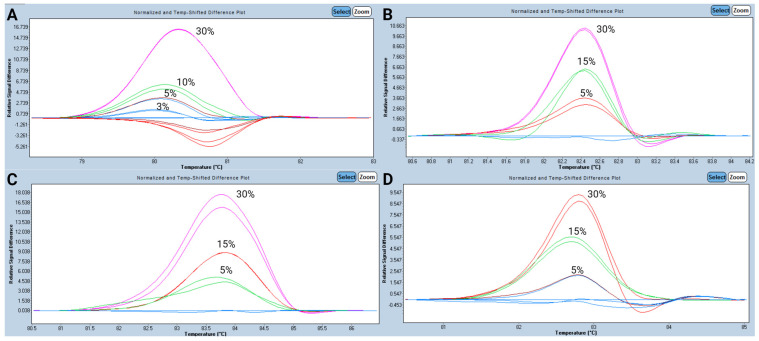
Difference plot analysis for a serially diluted positive sample with a known mutational burden. LOD analysis for *IDH1* (**A**). LOD analysis for *IDH2*^Arg140^ (**B**). C-LOD analysis for *IDH2*^Arg172^ (**C**). LOD analysis for *FLT3-TKD2* (**D**).

The lowest VAF detected by HRM was found at the 1:10 dilution for *IDH1* c.394C>T (p.Arg132Cys), corresponding to a 3% VAF (Figure 2A). *IDH2* c.419G>A (p.Arg140Gln), c.515G>A (p.Arg172Lys), and *FLT3-TKD2* c.2503G>T (p.Asp835Tyr) were detected up to the 1:6 dilution (5% VAF) (Figure 2B–D). Although the assay was able to detect *IDH1* c.394C>T (p.Arg132Cys) at a VAF as low as 3% (1:10 dilution), we decided to establish a common LOD for all determinations at 5% VAF.

### 3.3. Comparison of HRM with Sequencing Studies

**Figure 3:**
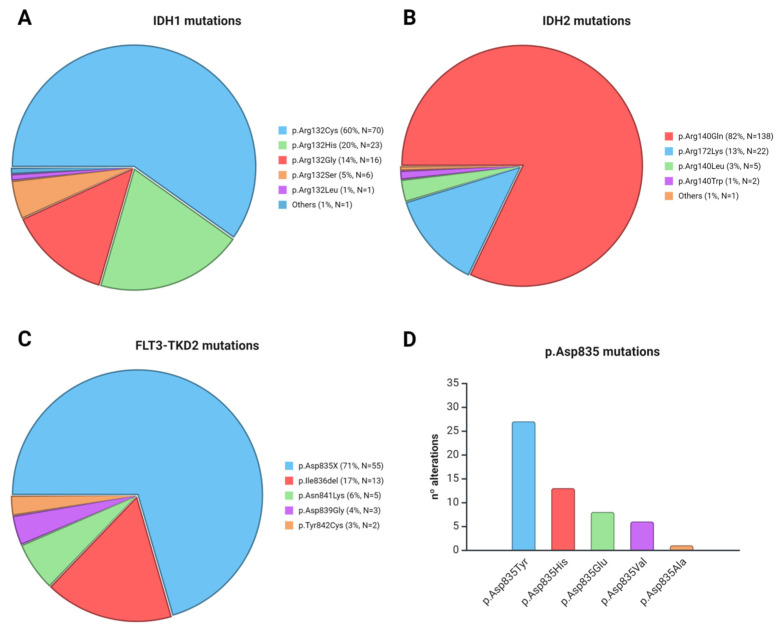
Mutational profile of *IDH1* (**A**), *IDH2* (**B**), and *FLT3-TKD2* (**C**,**D**) confirmed by Sanger and NGS sequencing.

HRM positive results were confirmed by direct sequencing, and all samples (positive and negative) were further analyzed by NGS. Among positive melt curve samples, we identified 70/117 c.394C>T (p.Arg132Cys) (60%), 16/117 c.394C>G (p.Arg132Gly) (14%), 23/117 c.395G>A (p.Arg132His) (20%), 6/117 c.394C>A (p.Arg132Ser) (5%), 1/117 c.395G>T (p.Arg132Leu) (1%), and 1/117 388A>G (p.Ile130Val) (1%) mutations affecting *IDH1* (Figure 3A). For *IDH2*, we found 138/168 c.419G>A (p.Arg140Gln) (82%), 22/168 c.515G>A (p.Arg172Lys) (13%), 5/168 c.419G>T (p.Arg140Leu) (3%), 2/168 c.418C>T (p.Arg140Trp) (1%), and one deletion at T146 (c.435del p.T146Lfs*15) (Figure 3B). The hotspot D835 in *FLT3-TKD2* was mutated in 55 out of 78 positive samples (71%). The specific mutations of codon 835 are shown in Figure 3D. Other alterations affecting this domain were found: 13/78 (17%) c.2508_2510del (p.I836del), 5/78 (6%) c.2523C>A (p.Asn841Lys), 3/78 (4%) c.2516A>G (p.Asp839Gly) and 2/78 (3%) c.2525A>G (p.Tyr842Cys) (Figure 3C).

No false positives (FP) were identified by NGS, while 12 false negatives (FN) (2 *IDH1*, 3 *IDH2*, and 7 *FLT3-TKD2*) were detected (Table 1). This resulted in a negative percent agreement (NPA) of 100% for all three genes, a positive percent agreement (PPA) of 98.32% for *IDH1*, 98.25% for *IDH2*, and 91.76% for *FLT3-TKD2* (Table 2). The overall percent agreement (OPA) was 99.85% for *IDH1*, 99.78% for *IDH2*, and 99.51% for *FLT3-TKD2*.

### 3.4. Turnaround Times

We conducted a comparative analysis of the turnaround time and cost between PCR-HRM and NGS for detecting *IDH1*, *IDH2*, and *FLT3-TKD2* mutations under standardized theoretical conditions. Both methods started from isolated genomic DNA to ensure a direct comparison. PCR-HRM demonstrated a significantly shorter turnaround time, delivering results within a median of 2 working days (range: 1–5 days), whereas NGS required a median of 12 days (range: 5–21 days), primarily due to longer library preparation, sequencing, and bioinformatics analysis times.

Regarding cost efficiency, PCR-HRM proved to be a substantially more affordable approach, with an estimated cost of EUR 20 per sample, while NGS was considerably more expensive at approximately EUR 500 per sample.

## 4. Discussion

In this study, we developed a novel PCR-HRM method for the screening of *IDH1*, *IDH2*, and *FLT3-TKD2* mutations. The analysis of more than 1000 AML samples showed reliable, sensitive, and low-cost results in a shortened turnaround time. This approach guarantees a rapid biomarker screening at diagnosis or relapse, which is critical for selecting suitable candidates for targeted therapies.

Mutational detection of key genes such as *IDH1*, *IDH2*, and *FLT3* can be achieved through various techniques, including restriction fragment length polymorphism (RFLP-PCR), real-time PCR, and direct sequencing [14,15]. However, many of these methods lack sensitivity or are time-consuming. In recent years, digital PCR (dPCR) has emerged as a powerful approach for mutation detection, offering unparalleled sensitivity and absolute quantification of mutant alleles. This technique is particularly useful for detecting low-frequency variants and monitoring minimal residual disease. However, dPCR remains more expensive and typically allows the identification of only a single alteration per assay, limiting its applicability for comprehensive mutational screening in routine clinical settings. A major advantage of the PCR-HRM over other techniques is its ability to detect canonical and non-canonical mutations across the entire hotspot region [23,24,25]. For instance, we identified one mutation outside *IDH1* R132 and seven mutations outside *FLT3-TKD2* Asp835/Ile836 codons. This is consistent with findings from Patel et al. (2011) [26], who explored the application of PCR-HRM for mutational screening in *IDH1* and *IDH2*, and Mahmoudi et al. (2021) [27], who used PCR-HRM for *FLT3* mutations. However, this study is the first to enable the simultaneous detection of *IDH1/IDH2* and *FLT3-TKD2* mutations in a single experiment, which enhances its utility in clinical settings. Other important advantages of this method are high-throughput capability and rapidity. PCR-HRM allows the analysis of all three genes in up to 12 samples simultaneously using 96-well plates in 3 h.

The LOD achieved with PCR-HRM was 5%, which is more sensitive than conventional Sanger sequencing. PCR-HRM identified two alterations in *FLT3-TKD2* (c.2503G>T [p.Asp835Tyr]) with VAFs of 9.35% and 8.40%, respectively, that were not detected by Sanger sequencing due to their low VAF. Although NGS exhibited the highest analytical sensitivity, achieving a LOD of 1%, PCR-HRM is sufficiently sensitive for most clinical applications [28,29]. Additionally, compared to techniques such as dPCR, which offer even greater sensitivity, PCR-HRM remains an attractive alternative due to its simplicity, cost-effectiveness, and ease of implementation in routine diagnostics.

The PCR-HRM method demonstrated 99% PPA and 100% NPA for all three genes. Comparison with NGS results showed no false positives, which supports the high positive predictive value of the PCR-HRM method. False negative results (mutations detected by NGS but not by PCR-HRM) were only detected in samples with a VAF < 5%, below the LOD established for PCR-HRM. These findings are consistent with previous studies evaluating HRM-based approaches for mutation screening, further validating their accuracy and applicability in a clinical setting.

Despite its advantages, PCR-HRM has some limitations. The 5% LOD means that mutations present at very low allele frequencies may be missed, potentially impacting the detection of subclonal populations in AML, particularly in the context of minimal residual disease (MRD) monitoring. In such cases, NGS or dPCR may be preferable. Additionally, PCR-HRM cannot directly identify the specific nucleotide change without further validation by sequencing, which may be necessary in cases where non-canonical mutations are suspected.

From a clinical perspective, rapid genetic screening is crucial for AML management, particularly for the selection of targeted therapies such as IDH1/IDH2 inhibitors or FLT3 inhibitors. Given its speed and affordability, PCR-HRM can serve as a valuable first-line screening tool in routine diagnostics, especially in laboratories with limited access to NGS. Future improvements in HRM curve analysis algorithms and the integration of artificial intelligence for automated mutation calling could further enhance the performance and applicability of this method.

In conclusion, our study presents a robust and reliable tool for assessing the mutational status of *IDH1*, *IDH2*, and *FLT3-TKD2* in AML patients at diagnosis or relapse. The PCR-HRM assay is faster and less expensive than NGS, facilitating rapid analysis and providing crucial results with a shortened turnaround time. These attributes make our method an attractive option in the rapidly evolving landscape of targeted therapies, where fast genetic analysis is essential for effective treatment decisions. Nonetheless, the assay’s limit of detection (5%) must be taken into account, as it may lead to missed detection of subclonal variants. Future research should aim to improve the analytical sensitivity of HRM-based assays, explore their integration with automated mutation-calling algorithms, and validate this approach in prospective clinical trials to assess its impact on clinical decision-making.

## Figures and Tables

**Table 1:** Alterations identified by NGS and not detected in PCR-HRM analysis.

Gene	Exon	Nº of Patients	Nucleotide Change	Mutation Detected *	VAF (%) ^a^
*IDH1*	4	2	CGT>TGT	p.Arg132Cys	2.40%
			CGT>TGT	p.Arg132Cys	1.80%
*IDH2*	4	3	CGG>CAG	p.Arg140Gln	3.20%
			CGG>CAG	p.Arg140Gln	3.10%
			AGG>AAG	p.Arg172Lys	4.00%
*FLT3*	20	7	GAT>TAT	p.Asp835Tyr	2.50%
			GAT>TAT	p.Asp835Tyr	3.00%
			GAT>TAT	p.Asp835Tyr	1.40%
			GAT>TAT	p.Asp835Tyr	4.70%
			GAT>CAT	p.Asp835His	3.90%
			delGATATC	p.Asp835_Ile836del	4.20%
			AAC>AAA	p.Asn841Lys	2.20%

^a^ Variant allele frequency obtained by NGS. * Variants not detected by PCR-HRM but identified by NGS (false negatives) corresponded to low-frequency variants with VAFs below the established LOD for the HRM assay (5%), consistent with the expected performance limitations of the method.

**Table 2:** Positive percent agreement and negative percent agreement of PCR-HRM.

Method	Gene	PPA, %[TP/(TP+FN)]	95% CI, %	NPA, %[TN/(TN+FP)]
HRM vs. NGS	*IDH1*	98.32[117/117+2]	97.64–99	100[1244/1244+0]
	*IDH2*	98.25[168/168+3]	97.41–98.82	100[1244/1244+0]
	*FLT3*	91.76[78/78+7]	90.33–93.19	100[1341/1341+0]

True positive (TP) refers to a known mutation expected to be found on the reference genome and that has actually been identified and called experimentally after sequencing. False positive (FP) refers to a called mutation that is not expected to be found when compared with the reference genome. True negative (TN) refers to any genomic position analyzed by the method that matches the base on the reference genome, whereby no mutation is expected to be found. Finally, a false negative (FN) refers to a position where a mutation is expected to be found but, after analysis, this variant has not been detected.

## Data Availability

Data are available upon reasonable request to barragan_eva@gva.es.
